# The Malnutritional Status of the Host as a Virulence Factor for New Coronavirus SARS-CoV-2

**DOI:** 10.3389/fmed.2020.00146

**Published:** 2020-04-23

**Authors:** Matteo Briguglio, Fabrizio Ernesto Pregliasco, Giovanni Lombardi, Paolo Perazzo, Giuseppe Banfi

**Affiliations:** ^1^IRCCS Orthopedic Institute Galeazzi, Scientific Direction, Milan, Italy; ^2^Health Management, IRCCS Orthopedic Institute Galeazzi, Milan, Italy; ^3^Department of Biomedical Sciences for Health, University of Milan, Milan, Italy; ^4^Laboratory of Experimental Biochemistry and Molecular Biology, IRCCS Orthopedic Institute Galeazzi, Milan, Italy; ^5^Department of Athletics, Strength and Conditioning, Poznań University of Physical Education, Poznań, Poland; ^6^Post-operative Intensive Care Unit & Anesthesia, IRCCS Orthopedic Institute Galeazzi, Milan, Italy; ^7^Faculty of Medicine and Surgery, Vita-Salute San Raffaele University, Milan, Italy

**Keywords:** nutritional status, coronavirus, SARS-CoV-2, COVID-19, infections, virulence, host pathogen interactions, quality of health care

## The Spill Out of SARS-CoV-2

An outbreak of viral pneumonia was reported in Wuhan, China, at the end of December 2019, and subsequent sample analyses discovered the involvement of a new strain of coronavirus (SARS-CoV-2), which belongs to the same family of single-stranded enveloped RNA viruses that caused the emergences of SARS-CoV in 2003 and MERS-CoV in 2012. Symptoms of COVID-19 (SARS-CoV-2 syndrome) may occur within 2–14 days after exposure and can lead to difficulties in cilium beating of airway cells and to alveolar damage (1). Infected patients experience mild to severe manifestations, such as fever, dry cough, dyspnoea, abdominal pain, and diarrhea. Most cases resolve rapidly, but the infection can still be fatal in about 3% of cases (2). Much like MERS or the coronavirus that infects pigs, the enteric affections can be prominent (3, 4), possibly leading to the loss of absorptive potential. Just a few weeks after its discovery, the COVID-19 has been considered a serious worldwide threat. At the time of writing, Italy is the worst-hit country with 97,689 confirmed cases and 10,781 total deaths (WHO COVID-19 Situation report 70, 30 March 2020). Preliminary data suggest that male older adults and subjects with immune dysfunctions might be more susceptible to the worse viral disease, but there is a need to further investigate the virulence factors. One of the factors most discussed is the malnutritional status of the host, but most of the beliefs are anecdotal. On the other hand, strong evidence supports the notion that any infection outcome is highly dependent on the nutritional status of the host since viruses subject the host's body to a considerable energetic effort to sustain costly defenses. If a previous malnutritional status exists, or if no nutritional care is provided, the host easily encounters the emptying of body reservoirs with increased harm caused by the virus. A possible link between the nutritional status of the host, the virulence of SARS-CoV-2, and the clinical outcome of COVID-19 needs to be discussed.

## The Host Abilities Against Infections

A distinction should be made between the susceptibility to developing a symptomatic infection, from now on referred to as “first-line host ability,” and the fighting potential, referred to as “second-line host ability.” From the perspective of infectious diseases, the first-line host ability is expressed by its immunocompetence, which is in turn uttered by the nutrient intake-requirement balance. A malnutritional status refers to any balance deviation, including the general excess, insufficiency, or single-nutrient deficits. The second-line host ability is expressed by the endurance or ability to persist in fighting the infection. For SARS-CoV-2, it can be assumed that the healthier is the nutritional status of the host, the higher are the first-line host abilities, the lower is the susceptibility to COVID-19, the lower is the virulence of SARS-CoV-2, and, thus, the longer the host will endure in the fight. This transitive relation is not necessarily assumable for all pathogens. Concerning parasitic infections, well-nourished subjects may offer a wealthier environment to developing parasites than malnourished individuals (5), but they can also afford investments to endure in the fight, still having the upper hand on the infection outcome. Whatever the nature of the susceptibility to viral infections, second-line host abilities are based not only on the ability to support an adequate immune response but rely also on the body's ability to support an extensive controlled catabolic cytokine flow. Once infected, the nutritional reservoirs have been shown to influence outcomes in many diseases, comprising the immunodeficiency virus, the influenza virus, or pneumonia (6, 7). The within-host reservoirs depend on the external environment (8); the highest resources should exist in hosts living in the wealthiest environments. Regrettably, even the wealthiest countries present high rates of deficiency syndromes.

## The Host Reactions Against SARS-CoV-2

Both first- and second-line host abilities are necessary to heal from SARS-CoV-2 infection. Once the virus gets inside the airways through respiratory droplets, it infects local cells and evokes the host immune response. Mild symptoms of COVID-19 may be triggered by a local inflammation limited to the lungs that should resolve quickly. Asymptomatic individuals have been reported to have no high fever (no increased expenditure) and no SARS-CoV-2-derived gastrointestinal symptoms that could have affected dietary intakes (9). The immunocompetent host response in non-severe cases recruits immune cell populations, such as CD4/CD8 T cells and antibody-secreting cells together with specific immunoglobulin (Ig)M and IgG SARS-CoV-2-binding antibodies (10). Basic treatments comprise intravenous antibiotics, antiviral therapy, antifungal medications, systemic glucocorticoids, and interferon. In cases with comorbid conditions, such as cardiovascular diseases and diabetes (11), there may be a basal immune dysfunction since the elderly and sick are often malnourished. If the immunoincompetence fails to control the SARS-CoV-2 or the virus replicates faster than expected, a severe inflammatory condition then arises and spreads to other organs together with the virus. Worsened patients show lymphopenia, cytokine storm (12), and multiple organ failure (13). These biochemical signs together with the decrease in CD4 T cells are a common feature in patients with COVID-19 and might be a critical virulence factor (14). The intestines may be particularly suitable for viral proliferation, as gut tropism in not unusual for coronaviruses. The host's ability to endure may depend on energy-nutrient intakes, which may be hampered by gastrointestinal symptoms and the hypermetabolism. Higher rates of nausea, vomiting, and diarrhea were observed in severe COVID-19 patients, which appear to be more likely to have anorexia (15). The prevalence of malnutrition (probably hyponutrition) was 3% among the deceased vs. 0% among survivors (16). Healthy body reservoirs, early adaptive immune potential, and nutritional care may indeed be associated with better outcomes from COVID-19.

## The Disabilities of Malnourished Individuals During Infections

“Malnutrition is the primary cause of immunodeficiency worldwide” (17) and affects both the innate and adaptive immune responses (18) that should inhibit viral proliferation. Chronic diseases, which have been recognized as virulence factors for severe COVID-19, are often comorbid with protein-energy malnutrition (also known as disease-related malnutrition), which is known to impair immune cell activation (19, 20), thus allowing longer viral persistence and increased trafficking of inflammatory cells to lungs (21). The basal immunoincompetence (22) can be further aggravated upon infection (23). Insufficient protein intakes may lead to nutrition-related sarcopenia. The concomitant excess of adiposity has been defined as “sarcopenic obesity” and carries issues of both conditions. Increased body fat sustains a systemic low-grade inflammation, primarily because of the leptin-induced CD4 T-cell function that increases autoimmunity (24). Basal T cells are more prone to exhaustion in obese subjects (25) who may therefore be more exposed to SARS-CoV-2 proliferation, as occurs with the herpes simplex virus (26). In fact, exhausted T cells exhibit poor effector function, proliferation, and cytotoxicity (27). During the 2009 pandemic caused by the influenza A (H1N1)pdm09 virus, obesity was found to be a virulence factor for a more severe outcome (28) much like for respiratory infections (29). Micronutrient deficiencies are also a rising issue among malnourished subjects. Vitamins have a role in the proper functioning of both the innate and adaptive immune responses, with vitamin D and A being the main actors (30). For instance, vitamin D is important for the proper functioning of antibody-secreting cells (31) and vitamin A sustains T-cell proliferation (32). The immune dysfunction in hyponutritional statuses can be linked to these deficiencies alike the excess of feeding, which is often associated with a monotonous diet and therefore low in sources of vitamins. A plethora of other micronutrients is known to have a role in the immunocompetence of the host against infections, including B vitamins, vitamin C, vitamin E, iron, selenium, and zinc (33), with malnourished individuals often suffering from the most. Malnutritional statuses carry less endurance to survive from severe COVID-19. Hypermetabolism and excessive nitrogen loss are factors known to be associated with infective states, and malnourished individuals are therefore disadvantaged because of the lesser body reserves. For instance, infected mice fed with lesser proteins, iron, and zinc than the optimal requirements were found to encounter a significant decrease in both weight and effector CD4 T cells vs. normal nourished animals (34).

## Nutritional Care in COVID-19

If the patient had a good nutritional status before infection, then body reserves and basal dietary intake would assure the coverage of costly immune defenses in mild conditions. If a malnutritional status was present, which is very common among older adults, then increased requirements should be provided since the infection is expected to be protracted (35). Mild cases might experience a loss of appetite often accompanied by insomnia, nausea, vomiting, and reduced oral intakes, thus further compromising the basal poor nutritional. Even subacute malnourished patients are more prone to adverse events than healthy counterparts upon hospital admission (36). Once mechanical ventilation, extracorporeal membrane oxygenation, and renal-replacement therapy have been introduced, parenteral nutrition is the sole option. Severe cases with fever have increased energy expenditure and requirements for each degree of temperature increase. The usage of muscle-derived amino acids for immune protein synthesis increases whole-body glucose and nitrogen excretion, with a significant energy cost of immune upregulation (37). Unfortunately, the increased adiposity of obese individuals is not effectively used during infections (38), and the breakdown of the already poor muscle mass can have severe consequences. Similar metabolic consequences are seen in older trauma patients, with the malnourished subjects being the most at risk of adverse clinical outcomes (39). If energy and protein requirements are met, then the emptying of body reservoirs may be avoided, and the immune response may be sustained. Once full-blown, COVID-19 patients should be supported with proper nutrition aimed at delivering adequate proteins (1.5–2.0 g/kg/day likely needed), energy (105–160 kj/kg/day or 25–40 kcal/kg/day), vitamins, and trace elements. Nutrition should be titrated up to meet higher requirements because the delivery of the highest energy during the initial phase may be counterproductive. Guidelines for polymorbid patients should be followed (40–42). Partial isocaloric replacement of carbohydrates with lipids may be considered to reduce the production of CO_2_ by 30% per caloric unit (43).

## Conclusion

**Figure 1:**
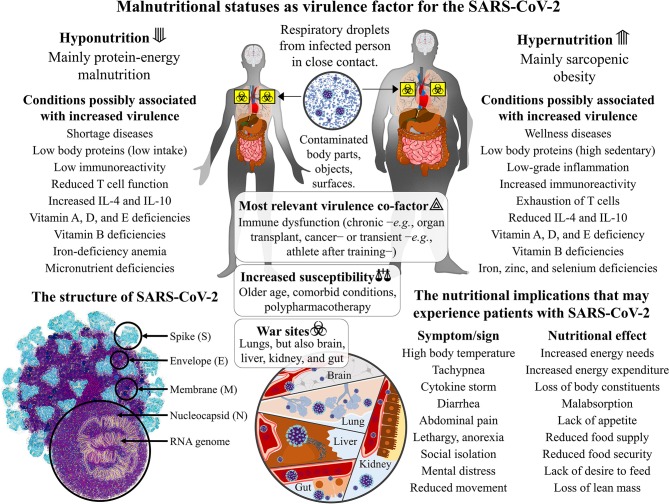
The SARS-CoV-2 virulence and the malnutritional status of the human host: immune-based dysfunctions in hypo- and hypernutrition. The severe acute respiratory syndrome coronavirus that was discovered in Hubei province, China, at the end of December 2019 (SARS-CoV-2) is a single-strand positive-sense RNA virus with the encoding potential of four structural proteins: the spike (S), the envelope (E), the membrane (M), and the nucleocapsid (N). It spreads through respiratory droplets of infected patients that can deposit on body parts and fomites. The basal immune dysfunction that exists in protein-energy malnutrition and sarcopenic obesity can make individuals more susceptible to SARS-CoV-2 contraction and affections. Other than the collapse of alveoli and respiratory failure, the coronavirus replication leads to systemic consequences in the brain, liver, kidneys, and gut. Once affected, malnourished individuals will have fewer body reservoirs and immune potential to fight for recovery.

In the current pandemic panorama of SARS-CoV-2, the link between nutrition and virulence takes a predictable turn. On one hand, many opportunists boast dietary plans against SARS-CoV-2, and, on the other hand, there is the sellout of dietary supplements that boost the immune system. In Italy, many instances of fake news have circulated on social networks, and many pharmacies have exposed signs that state: “Masks sold-out but vitamin C available.” In these times of fear and confusion, speculations should be disciplined. Nonetheless, a greater understanding of the link between nutrition and SARS-CoV-2 is needed, as the pathogen fitness may also depend on the host available resources (44). Future studies should focus on the transmission potential of malnutritional statuses. In the past, these conditions were suggested to negatively influence the transmission of alphaviruses to other hosts (45). Since most of infected cases are asymptomatic, the spreading of the virus is much easier than the previous coronaviruses (46). Yet, the risk of contracting SARS-CoV-2 does not depend on the individual's nutritional status but on the degree of contact with the pathogen. Whether the coronavirus exposure develops into a true infection might contrariwise depend on the individual's first-line abilities, and, regrettably, malnutrition is a common occurrence that afflicts many older adults in China (47) and Italy (48), both having been heavily afflicted by the highest number of deaths. It is clear that the segment of population most at risk of SARS-CoV-2 infection is the elderly, with frailty (49) and older age (50) being well-known predictors of a negative outcome in acute care settings. Intensive clinical monitoring at admission with subsequent tailored nutritional care is needed for COVID-19 patients, especially those with co-existing chronic conditions or medications that could further aggravate the nutritional status (51). To conclude, there are several main nutritional issues to consider when fighting COVID-19 (see Figure 1 for details). A malnutritional status is associated with immune dysfunction. Malnourished individuals may be more susceptible to SARS-CoV-2 infection. Subjects with COVID-19 often become malnourished. Nutritional support is vital in severe COVID-19 patients.

## Author Contributions

MB formulated the hypothesis and wrote the first draft of the manuscript. FP, GL, PP, and GB revised the first draft and contributed to manuscript sections. All authors contributed to manuscript revision, read, and approved the submitted version.

## Conflict of Interest

The authors declare that the research was conducted in the absence of any commercial or financial relationships that could be construed as a potential conflict of interest.
